# Cellular Therapies in Solid Organ Allotransplantation: Promise and Pitfalls

**DOI:** 10.3389/fimmu.2021.714723

**Published:** 2021-08-30

**Authors:** Brian I. Shaw, Jeffrey R. Ord, Chloe Nobuhara, Xunrong Luo

**Affiliations:** ^1^Department of Surgery, Duke University, Durham, NC, United States; ^2^School of Medicine, Duke University, Durham, NC, United States; ^3^Department of Medicine, Division of Nephrology, Duke University, Durham, NC, United States

**Keywords:** allotransplantation, donor specific transfusion (DST), donor specific antibodies, mesenchymal stem cell, sensitization, allosensitization, tolerance

## Abstract

Donor specific transfusions have been the basis of tolerance inducing protocols since Peter Medawar showed that it was experimentally feasible in the 1950s. Though trials of cellular therapies have become increasingly common in solid organ transplantation, they have not become standard practice. Additionally, whereas some protocols have focused on cellular therapies as a method for donor antigen delivery—thought to promote tolerance in and of itself in the correct immunologic context—other approaches have alternatively focused on the intrinsic immunosuppressive properties of the certain cell types with less emphasis on their origin, including mesenchymal stem cells, regulatory T cells, and regulatory dendritic cells. Regardless of intent, all cellular therapies must contend with the potential that introducing donor antigen in a new context will lead to sensitization. In this review, we focus on the variety of cellular therapies that have been applied in human trials and non-human primate models, describe their efficacy, highlight data regarding their potential for sensitization, and discuss opportunities for cellular therapies within our current understanding of the immune landscape.

## Introduction

Presently, solid organ allotransplantation is hampered by poor outcomes due to the nonspecific effects of immunosuppressive medications, especially calcineurin inhibtors (1, 2). Indeed, improvements in long term graft outcomes in both liver and kidney transplant have been minimal and disappointing in the past 20 years (3–5). In spite of this, there has been a consolidation of immunosuppressive management, with the vast majority of patients now managed on a calcineurin-based regimen (6).

**Figure 1:**
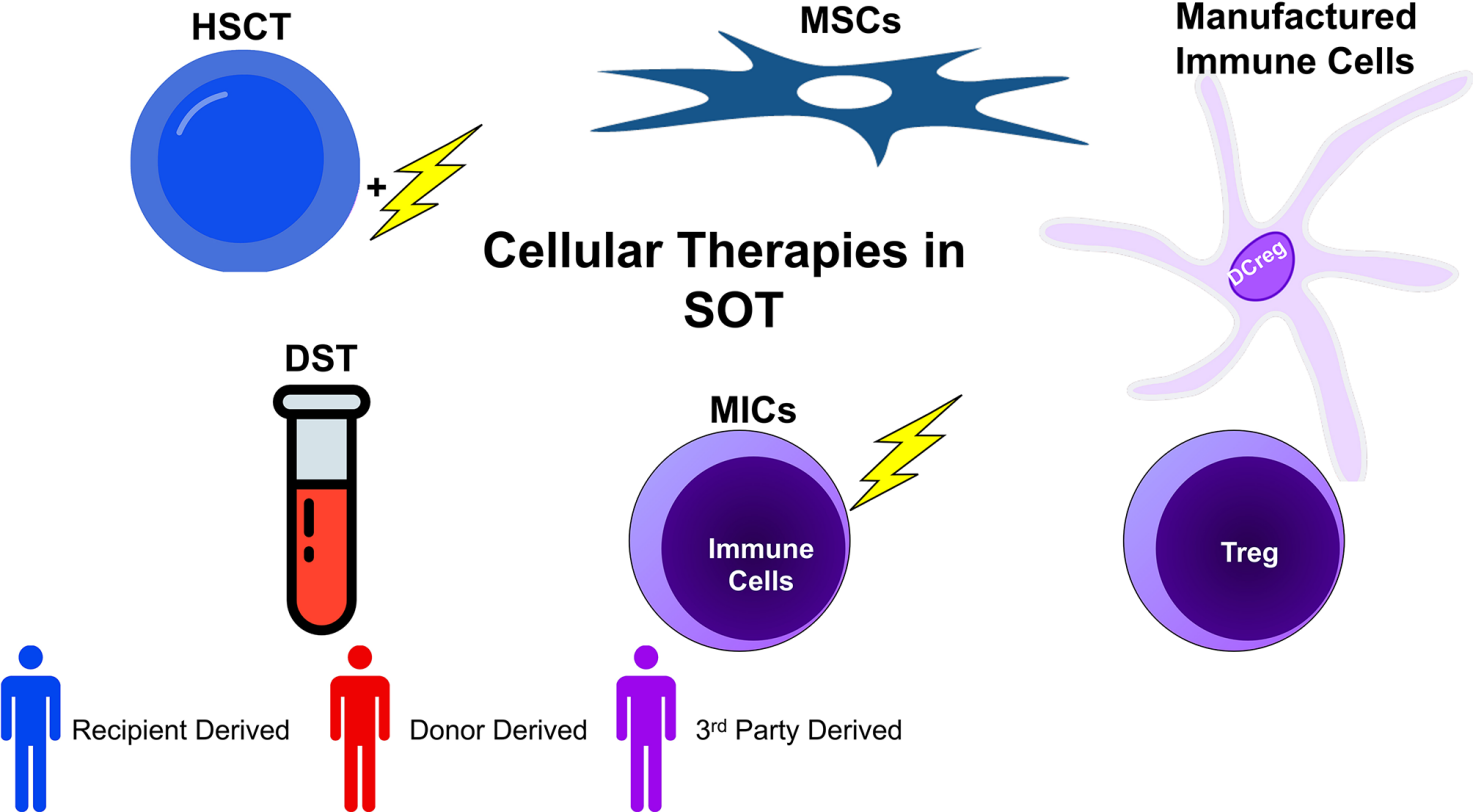
Cellular therapies in transplantation can be broadly categorized into five groups: donor specific transfusions (DSTs), hematopoietic stem cell transplant (HSCT), mesenchymal stem cell based therapy (MSC), manufactured immune cell therapy (using various regulatory cells), and modified immune cell (MIC) therapy. These broad categories can be further subdivided by the source of the cells. Whereas recipient derived cells carry no risk of sensitization (or very little), donor derived cells inherently may sensitize the recipient. Additionally, 3^rd^ party cells, depending on their genetic similarity with donor and recipient, may or may not be sensitizing. When evaluating cellular therapies, both the function and origin of cells are of import.

In a distinct vein, there has long been an interest in the ability to specifically modify the immune response to donor antigens. This research has focused on utilizing donor derived biological products across a number of cell types and preparations to condition the immune system to accept the allograft and is based, conceptually, on the work of Sir Peter Medawar (7). In spite of Medawar’s early experiments, however, the ability to not simply blunt the total immune response but rather specifically inhibit the response to the donor has been elusive. Indeed, it has been haunted by the fact that specific inhibition requires that an immune system be exposed to the antigens which it may respond to, giving rise to a risk of sensitization. The use of regulatory cell therapies may be a way to avoid this, but they remain incompletely explored. An overview of cellular therapies currently under investigation may be found in **Figure 1**.

In the present manuscript, we will review the progress made in the application of donor specific cellular therapies starting with an examination of early evidence. We will further describe the multiple cellular strategies currently being investigated in clinical trials including the combination of donor specific transfusion (DST) with novel immunosuppressive regimens, modified cellular therapies, and specific cell type based therapies. Finally, we will examine the future landscape of donor directed therapies and the unmet research need. 

## The Origins of Cellular Therapy in Transplantation

Few experiments in transplantation are as well-known as those of Sir Peter Medawar. In his seminal set of studies, Dr. Medawar built on the work of Dr. Ray Owen (8) and demonstrated that intra-embryonic injection of cells from future skin donors led to tolerance among a subset of mice (7). Ultimately, Medawar was awarded the Nobel Prize for this work and it remains the theoretical basis for many tolerance inducing protocols today. A few specific aspects of the experiment are worth noting. First, the injections occurred early in the development of the mice; therefore, the thymus was robust and actively educating T cells. Thymic development continues to adolescence when involution begins (9). Therefore, these injections in the fetal period had the advantage of potentially utilizing the positive and negative selection programs of the thymus to abrogate responses to the alloantigens.

Additionally, they utilized a mix of donor cells from the “testis, kidney, and splenic tissue” (7). Here, the splenic tissue used suggests a high proportion of B cells as well as professional antigen presenting cells (APCs) which are known to express higher levels of major histocompatibility complex (MHC) class II as well as class I, which is constitutively expressed. That is, there were a variety of antigens expressed by these tissues and therefore available to the recipients. Taken together, these details suggest that both the character of donor derived infusions and the overall immunologic context (in this case most modified by the pre-natal environment) may determine the fate of the immune response.

Though his experiments were conceptually very important, the clinical reality of early transplantation was more complex and the application of cellular products of as treatment remains elusive (10). As adults were the first recipients of transplanted organs, the selective programs of the thymus were less available. Early experience with DST showed the now predictable result of allosensitization leading to the inability to transplant and a divisive discussion within the transplant community regarding the benefit of transfusion pre-operatively (11). Still, results were heterogenous: some early animal models hinted at the benefit of DST (12) and a number of early studies indeed showed that non-leukoreduced transfusions in general (not donor specific) appeared to lengthen graft survival, though the mechanism was unknown (13, 14).

Salvatierra and colleagues were some of the first to specifically evaluate the role of DST on graft survival in a systematic way (15). In this seminal work, they showed that while 1/3 of patients became sensitized (as measured by persistently high levels of alloantibody), 2/3 of patients were able to be transplanted. These patients had improved graft survival compared to similarly mismatched controls (94% *vs*. 56%). Despite a high degree of donor responsiveness as measured by mixed-lymphocyte reaction (MLR), patients who received DST had lower rates of acute rejection in the first 3 months using similar immunosuppression (44% *vs*. 82%). From these experiments, it was difficult to determine the exact differentiating factor which caused sensitization in some and tolerogenic effects in others. However, the authors did show that all patients who became sensitized with DST had also received multiple other blood transfusions. In this instance, the treatment was standardized (non-frozen, non-leukoreduced DST) but the context, potentially measured by the amount of 3^rd^ party (i.e., neither donor nor recipient) blood transfusions, may have modified the ultimate immune response to donor antigen. Concomitantly, experimentation in mouse models showed similar results with improved skin graft survival with DST but not with 3^rd^ party transfusion (16).

Over the next decade, several studies continued to illustrate the principles by which DST may be beneficial. First, evidence showed that the salutary effect of DST was most apparent in human leukocyte antigen (HLA) mismatched recipients, whereas it had little effect in fully haploidentical recipients (17). This was predictable as HLA matched recipients may have fewer antigens to tolerize to and therefore the effect would be of a lower magnitude. Additionally, it is worth noting that even in the Salvatierra study, the graft survival of the high reactivity MLR group was worse than that of the low reactivity MLR group (which did not receive DST), even with the addition of DST (15). Other studies suggested that at least some degree of HLA similarity was needed for improved graft survival with DST (18). Further mechanistic studies were also undertaken around this time which showed that it was possible that APC-depleted DST were more effective at inducing tolerance (19), suggesting that the type of cell was of import. However, due to the continued risk of sensitization, DST fell out of favor throughout the 1990s and early 2000s, culminating in a meta-analysis in 2010 which concluded that the risks of DST outweighed the benefits in the modern immunosuppressive era (20).

## Combining DST and Costimulation Blockade

One area where DST continues to be investigated is in conjunction with costimulation blockade. Early murine experiments showed indefinite islet graft survival when DST with small lymphocytes (with or without T-cell depletion) was combined with anti-CD154 (21) or when DST with splenocytes was combined with CTLA4Ig (22) or anti-CD154 (23). Elegant mechanistic experiments showed that these effects may be mediated by deletion of alloreactive cells (24), the production of allospecific suppressor cells (25), or the induction of anergy (26) and appeared to depend at least in part on indirect presentation of donor antigens by recipient MHCs, whereas direct presentation was dispensible (27). Indeed, improved outcomes were achieved using DST in conjunction with rapamycin and anti-CD154 in a rhesus macaque model of skin transplantation, compared to rapamycin and anti-CD154 alone (28). This prompted further trials which showed operational tolerance in kidney transplant in both rhesus macaque (29) and cynomologous (30) models. Trials using belatacept based costimulation blockade, rapamycin, and DST in the form of donor bone marrow have been completed with many patients able to be maintained on belatacept monotherapy with excellent renal function outcomes (31, 32). In these studies, alemtuzumab—an anti-CD52 monoclonal antibody which is a potent depletional induction agent which causes near immediate (33) and long lasting lymphopenia, up to 6 months (34)—was used. This potentially allowed for remodeling of the immune system during its reconstitution (35). Of note, a total of n = 5 (12%) of patients on this protocol developed *de novo* DSA after transplant, though it is unclear what clinical significance these antibodies have.

## DST Using Modified Cellular Products

Another distinct line of investigations has attempted to deliver donor antigen itself in a more tolerogenic manner. Beginning with multiple early studies in mice, Luo and colleagues have shown that pre-treating cellular infusions (in this case splenocytes) with ethylene carbodiimide (ECDI) leads to long term tolerance in a high proportion of mice undergoing islet transplantation into the kidney capsule (36). Of note, they showed that this effect was donor specific, with no prolongation of 3^rd^ party grafts. Multiple findings from this first study are mechanistically interesting. First, they found that the ablation of Treg *via* an anti-CD25 antibody prior to ECDI-treated cell infusion abrogated tolerance induction but late (120 days after transplantation) depletion of Treg did not. Additionally, they showed that the PD-1 axis was necessary for tolerance induction.

Following on from these studies, it was demonstrated that ECDI treated splenocytes could condition tolerance in murine cardiac allografts (37). Additionally this tolerance could be achieved not only with splenocytes but also culture-expanded B cells or cryopreserved cellular products and could be combined with clinically relevant immunosuppressive regimens (38). Mechanistically, these infusions cause an expansion of myeloid derived suppressor cells (MDSCs) both at the site of the allograft (37) and systemically (39) which appear to be important in abrogating deleterious T-cell responses. ECDI treatment leads to apoptosis of the infused cells and therefore presentation of donor antigen in an apoptotic context. This should attenuate any inflammatory immune response and is generally termed efferocytosis—the clearing of apoptotic cells in a non-inflammatory manner (40). Specifically, it has been demonstrated that this anti-inflammatory program is mediated by the receptor tyrosine kinase MerTK which participates in the phagocytosis of apoptotic cells in an allotransplantation context *via* suppression of IFN-α signaling and subsequent expansion of MDSCs (41).

Recently, this strategy has also expanded into a non-human primate (NHP) model of islet cell transplantation, again, using splenocytes as the donor cell source supplemented by culture-expanded donor B cells from the peripheral blood. This lead to long term tolerance in a high proportion of recipients (42). Multiple experiments confirmed that there was a decrease in donor-specific T-cell response and an increase in the proportion of MDSCs. Of note, this protocol was not successful when primates had previous sensitization to donor antigens. However, in mice, ECDI treated splenocyte infusion when combined with rapamycin and anti-CD154 (MR1) led to prolonged survival in sensitized recipients relative to rapamycin and anti-CD 154 alone (43). Overall, ECDI treated cells represent one cellular therapy that is donor specific, that does not appear to cause sensitization, and may allow for the reduction or withdrawal of immunosuppression in select patients. Further work is needed to translate this therapy into vascularized grafts in NHP models in anticipation of human trials.

Another line of investigation that parallels the treatment of splenocytes with ECDI is the use of modified immune cells (MICs). Terness and colleagues initially observed that DCs treated with mitomycin C become tolerogenic in an *in vitro* context, potentially *via* downregulation of costimulatory molecules CD80/CD86 and ICAM-1. Intriguingly, T cells exposed to these DCs became stably tolerized to the antigens presented as these cells could not be restimulated after co-culture with these MICs (44). Further experiments showed that MICs prolonged graft survival in rat heart transplantation and again showed a phenotype of downregulated costimulatory molecules. Indeed, they achieved the same clinical effect using antibodies that coated DC costimulatory molecules and blocked the productive interaction of other cells with these DCs (45). They further extended these results from sorted DCs to whole PBMC in both rat and porcine contexts with improved graft survival with mitomycin C treated PBMC infusion (46). They also showed improved survival in a vascular composite allograft context in rats (47). Recently, they completed a trial in humans where they showed both safety of MICs derived from whole PBMC as well as specific inhibition of donor responses in patients treated with MICs (48). However, as this was a phase 1 study, patients were maintained on CNI for immunosuppression and no comparator group was examined. Of note, they also showed an increase in regulatory B cells and did not observe any sensitization in the study. In sum, modified cellular therapies are a promising but incompletely studied form of cellular therapy that may specifically inhibit the recipient response to donor. 

## Combined Hematopoietic Stem Cell Transplant and Solid Organ Transplantation

Another area where a form of DST continues to be investigated is in chimerism protocols. Early murine models showed tolerance could be achieved by the depletion of the recipient immune system followed by concomitant hematopoietic stem cell transplant (HSCT) and solid organ transplant from the same donor (49). Within a few years, this result had also been replicated in NHP (50). Mechanistically, this tolerance is thought to be due to chimerism (51). Though the outcomes were exciting, there was some hesitation as macrochimerism can lead to graft *vs*. host disease (GVHD), especially with the use of HLA-mismatched donors (52). Studies using combined HSCT in conjunction with solid organ transplant in humans were first pursued among patients with multiple myeloma who also qualified for kidney transplant. In these patients, they were conditioned using cyclophosphamide, anti-thymocyte globulin (ATG), and thymic irradiation. Though initial results with one (53), and then six subsequent patients (54) were encouraging, long term follow-up demonstrated GVHD (either acute or chronic) in 4/7 patients and the need for at least some immunosuppression in 3/7 patients (55).

In a more targeted way, the same group as above also performed combined HSCT and kidney transplant in five patients using a non-myeloablative regimen consisting of cyclophosphamide, thymic irradiation, and anti-CD2 monoclonal antibody, and a short course of cyclosporine. Four out of five patients were able to be weaned off all immunosuppression with stable graft function. Interestingly, the second patient on this protocol developed acute humoral rejection with early graft loss (day 10) in spite of a negative crossmatch, though none of the patients developed GVHD (56). Other groups have also attempted to achieve tolerance using combined HSCT and kidney transplant with variable success. One group has utilized total lymphoid irradiation and ATG for conditioning with a T-cell depleted HSCT infusion which has led to at least transient chimerism and the ability to withdraw immunosuppression in 16/22 (72% of patients) who were HLA-matched (57–59). However, none of the patients on this protocol who were HLA-mismatched have yet been able to undergo immunosuppression withdrawal. A final group has attempted HSCT with fludrabine, cyclophosphamide, and whole-body irradiation condition in conjunction with kidney transplant and shown the ability to withdraw immunosuppression in most patients that have stable chimerism, with some now up to 9.5 years off all immunosuppression (60–62). However, only 6/20 total patients and 6/15 who underwent weaning are currently still off immunosuppression. Though these protocols have been very successful in achieving tolerance in a small number of patients, the high morbidity and continued concern regarding GVHD has limited their widespread adoption. 

## A Shift to Cell Type Based Therapies—The Rise of Mesenchymal Stem Cells

As the field of transplantation shifted away from DST based strategies and early enthusiasm for non-CNI based therapies waned due to concerns about increased rates of acute rejeciton (63–66), there was a concomitant increase in interest in cellular therapies of specific cell types, especially ones that may be derived from the recipient themselves. One early stream of investigation targeted the use of mesenchymal stem cells (MSC). MSCs are heterogenous progenitor cells which can differentiate into mesodermal tissues, are adherent to plastic, and express certain cell surface markers. Of note, MSCs may be derived from many tissues but in practice are mostly isolated from bone marrow *via* culture methods that take advantage of the specific propensity of MSCs to adhere to plastic (67). Regardless, expansion of cells is required as the dose used is approximately 1.5 x 10^6^ cells/kg body weight (68). It should be mentioned that the preparations of MSCs do vary widely, and some groups have shown that both cryopreservation itself and the way in which MSCs are prepared after cryopreservation, for example, influences their immunosuppressive effect (69).

The first description of MSCs as immunosuppressive was in 2002 when investigators utilized MSCs in a skin transplantation model among baboons (70). They noted that MSCs inhibited MLRs to alloantigens and that MSC administration to baboons prolonged skin graft survival. Importantly, they also noted that the effect of MSCs was independent of their origin. That is, MSCs prolonged skin grafts regardless of whether the grafts were from the same donor as the MSCs or 3^rd^ party, suggesting a general immunosuppressive effect, not an allospecific one.

Mechanisms of MSC’s immunosuppressive effect are various and have been reviewed extensively in the literature (71). For the purposes of this review, the most salient mechanisms are the generation of tolerogenic APC. Early experiments showed that MSCs decreased ability of co-cultured dendritic cells (DCs) to stimulate T cells, including in an allostimulatory context (72). Further experimentation showed that this effect was in part due to the inhibition of DCs from entering the cell cycle (73) and potentially mediated by the soluble factors IL-6, IL-10, and hepatocyte growth factor (74). Additionally, MSC conditioned DCs have been shown to preference the generation of Treg *via* a CCL18 dependent mechanism (75). Indeed, taken together, these data together suggest that MSCs may ultimately change the context in which antigen is presented and therefore promote a tolerogenic phenotype. However, it is sobering to think that while MSCs may modify APCs to prevent a productive response to certain antigens, other cellular therapies may instead potentiate responses.

Consistent with the above mechanisms, further animal studies after Bartholemew’s initial description showed prolongation of graft survival regardless of origin of MSC. An early study in rat liver transplantation showed graft survival prolongation with MSC derived from donor, third-party, or syngeneic animals (76). Further studies in rat corneal transplant also showed prolongation of graft survival with third-party derived MSCs (77). Experiments in mouse models of heart (78) or kidney (79) transplantation showed prolongation of graft survival with the infusion of syngeneic MSCs when administered pre-transplant.

The first human trials of MSCs in transplantation were spurred on by safety in other fields (80, 81) and culminated in early clinical studies which showed the safety of MSC infusion among kidney transplant recipients (82, 83). Due to the nonspecific nature of the immunosuppressive effects of MSCs, early trials generally used autologously derived MSCs—thought to be the safest product—combined with kidney transplantation (84–86). In a randomized controlled trial setting, autologous MSC infusion with kidney transplantation and tacrolimus showed a lower incidence of acute rejection and opportunistic infection (83). However, the incidence of rejection in the standard therapy group was high (approximately 20%) and longer follow-up remains to be reported. Later trials in kidney (87, 88), liver (89, 90), and lung (91) transplantation utilized 3^rd^ party derived MSCs and demonstrated a similarly good safety profile.

One study did use donor derived MSCs which have the theoretical benefit of both nonspecific immunosuppression combined with antigen specificity. Again, the authors demonstrated a good safety profile and were able to use a lower dose of tacrolimus in the MSC group compared to the standard immunosuppression group (92). Of note, there was no assessment of the development of alloantibody in this trial. In the opposite manner, a study used MSCs which were mismatched to the recipient HLA *and* the donor HLA (i.e., they were no “repeated mismatches”) and showed good safety (93). This study did assess for sensitization to the specific 3^rd^ party alloantigen and did not observe it. Though one recipient had pre-existing allo-antibody to an MSC donor antigen, the antibody titer did not change with MSC infusion.

An interesting result from an early autologous MSC safety trial was the detection of an MSC infiltrate in a kidney graft when MSCs were administered 7 days after transplant (82). Though this did not lead to any production of antibody (the MSCs, again, were autologous) or lasting graft damage, there was a transient increase in creatinine. This may have been due in part to the proinflammatory environment of the recently implanted kidney. Indeed, a body of research exists that shows that innate signaling *via* molecules such as damage associated molecular patterns (DAMPs) (94) may modify the alloresponse. Similarly, these factors may be important when considering the infusion of dynamic cellular therapies.

One other important consideration of cellular therapies generally is their safety, which has been extensively reviewed previously for MSCs (95, 96). Important to transplant, prior studies have found that MSCs may promote a pro-coagulable state *via* the instant blood mediated inflammatory reaction (97), though administration directly into the bone marrow may mitigate this (98). Regardless, the clinical context of MSC therapy must be taken into consideration as this hypercoagulability may be deleterious to certain clinical conditions, for examples, in COVID-19 infeciton (99). In vascularized organ transplant, the potential for thrombosis of newly anastomosed vessels should lead to the tracking of these types of events when cellular therapies are utilized.

Overall, the data show that, while safe and slightly immunosuppressive in humans *in vivo*, MSCs do not themselves appear to condition for tolerance or cause drastic shifts in the recipient immune system. Indeed, a recent meta-analysis came to much the same conclusion (100). Of special note, present data do not suggest that MSCs are sensitizing, potentially due to their concomitant immunosuppressive effects, which raises the possibility of utilizing them as a delivery vector for alloantigen. 

## Other Cell Type Based Therapies—Treg and Beyond

Besides MSCs, many other cell types have been investigated. The largest study of regulatory cell based immunosuppressive products, the ONE study (101), was recently published. In this study, the safety of six different autologously derived cellular products was assessed using seven single arm studies that were harmonized for comparison. A single control arm was standard of care therapy for living donor renal transplant recipients using basiliximab induction, tapered steroid, mycophenolate mofetil, and tacrolimus. The six phase 1/2a interventional single arm studies consisted of two polyclonal Treg studies, two donor-antigen specific Treg studies, a tolerogenic DC study, and a regulatory macrophage study. As these were safety studies, no minimal graft outcomes were reported but all infusions were well tolerated and rejection events were similar between the control and interventional studies. Additionally, there were similar rates alloantibody production between the control and interventional studies. As all products were derived from recipients, the risk of sensitization was low, though the donor specific Treg were incubated with donor cells in order to achieve their specificity. A recent subset analysis of the natural Treg infusion data from this study has shown that these patients were more likely to be weaned to a monotherapy immunosuppression regimen (102). Still, the preparation of these cells is laborious and the appropriate timing of infusions remains unknown.

## Conclusions

Cellular therapies, especially those derived from donors, carry great potential but also great risk. Early studies showed that DST may have a salutary effect in certain patients, but an inability to determine who may benefit from these transfusions and the high degree of sensitization (up to 1/3 of patients) led clinicians to abandon these therapies. A shift towards specific cell type based therapies has been productive in improving the field’s ability to manufacture and deliver consistent therapies but has yet to revolutionize the way in which we approach immunosuppression. Modified cellular therapies are on the horizon and represent an exciting development that is grounded in long standing understanding of mechanisms of the immune response. Combinations of DST with costimulation blockade have emerged as a promising approach. However, questions remain regarding sensitization, long-term outcomes, and the pool of patients appropriate for costimulation blockade-based therapy. Hematopoietic stem cell transplant in conjunction with solid organ transplant is less clinically applicable due to the morbidity involved with conditioning, even in the absence of high rates of GVHD. Regardless of the cellular therapies pursued, there are key characteristics that these therapies must possess to be most useful (summarized in **Table 1**).

**Table 1:** Ideal properties of a cellular infusion and whether current therapies meet those criteria.

Characteristic	Explanation	DST	HSCT	Modified immune cells	MSC	Manufactured regulatory cells
Readily available	The ideal infusion would be readily available for administration at a reasonable timeframe with relation to transplant. E.g., Autologous therapies that could be derived over the course of a workup, donor derived therapies that are amenable to generation even in a time sensitive deceased donor context, or 3^rd^ party infusions that could be used “off-the-shelf.”	+/-	+/-	+/-	+	–
Specific to donor	Through either modification of the recipient immune system or delivery of donor antigens in a tolerogenic manner, the cellular infusion would specifically inhibit the recipient immune system from responding to donor antigens.	+	+	+	+/-	–
Lack of sensitization	Infusions should not cause sensitization (i.e., the generation of productive anti-HLA antibody) to either the donor or any other individual.	–	–	+	+ (autologous)	+
Effective regardless of sensitization status	The ideal infusion would be able to not only prevent the generation of responses to donor in recipients that were naïve to their donor but also delete pre-existing responses in order to expand the donor pool for highly sensitized individuals.	+/-	+/-	+/-	+	Unknown
Does not interfere with standard immunosuppression	Infusions should be compatible with a wide variety of immunosuppressions such that patients may be placed on the most appropriate therapy for their clinical condition	+/-	–	+	+	Unknown

(+), does meet the requirement; (-), does not meet the requirement; DST, donor specific transfusion; HSCT, hematopoietic stem cell transplant; MSC, mesenchymal stem cells.

Further phase 1 studies should continue to follow the example of the ONE study and others who attempt to gain as much information as possible with the most parsimonious control group. Additionally, further investigations into modifications of donor cells may be one way to overcome the significant hurdle that is sensitization. Transplantation is the original precision medicine, with HLA matching and crossmatch testing ensuring that organs implanted function appropriately and for the longest possible time. Cellular therapies ought to consider this precision in their development given the cost and potential for adverse events. In the future, the extent to which a therapy can inhibit a donor specific response relative to its overall immunosuppressive effect may become the most important metric, rather than a simplistic view which preferences only the inhibition of rejection episodes.

## Author Contributions

BS drafted the manuscript. JO performed critical review and revision of the manuscript. CN performed critical review and revision of the manuscript. XL performed critical review and revision of the manuscript and oversaw the drafting process. All authors contributed to the article and approved the submitted version.

## Funding

BS was supported by NIH grant R38 AI140297.

## Conflict of Interest

The authors declare that the research was conducted in the absence of any commercial or financial relationships that could be construed as a potential conflict of interest.

## Publisher’s Note

All claims expressed in this article are solely those of the authors and do not necessarily represent those of their affiliated organizations, or those of the publisher, the editors and the reviewers. Any product that may be evaluated in this article, or claim that may be made by its manufacturer, is not guaranteed or endorsed by the publisher.
